# The impact of Tsunamis on land appraisals: Evidence from Western Japan

**DOI:** 10.1371/journal.pone.0248860

**Published:** 2021-04-06

**Authors:** Yasuhiro Sato, Keita Shiba

**Affiliations:** 1 Faculty of Economics, University of Tokyo, Tokyo, Japan; 2 Research Center for Social Systems, University of Shinshu, Nagano, Japan; Universitat Jaume I, SPAIN

## Abstract

This paper estimates the impact of the tsunami caused by the Great East Japan earthquake on land appraisals of various locations outside of directly damaged areas. The focus is on locations that are expected to be extensively damaged by a tsunami if the Nankai Trough earthquake occurs. We use the DID and DDD approaches and show that locations with low elevation and close to the sea experienced decreases in appraised land prices compared to locations with high elevation and far from the sea. Especially, locations with less than 3.6m elevation and within 1.46km of the coastline experienced significant decreases in appraised land prices. This result implies that people have changed their location preferences regarding elevation and distance from the sea.

## 1. Introduction

**Fig 1:**
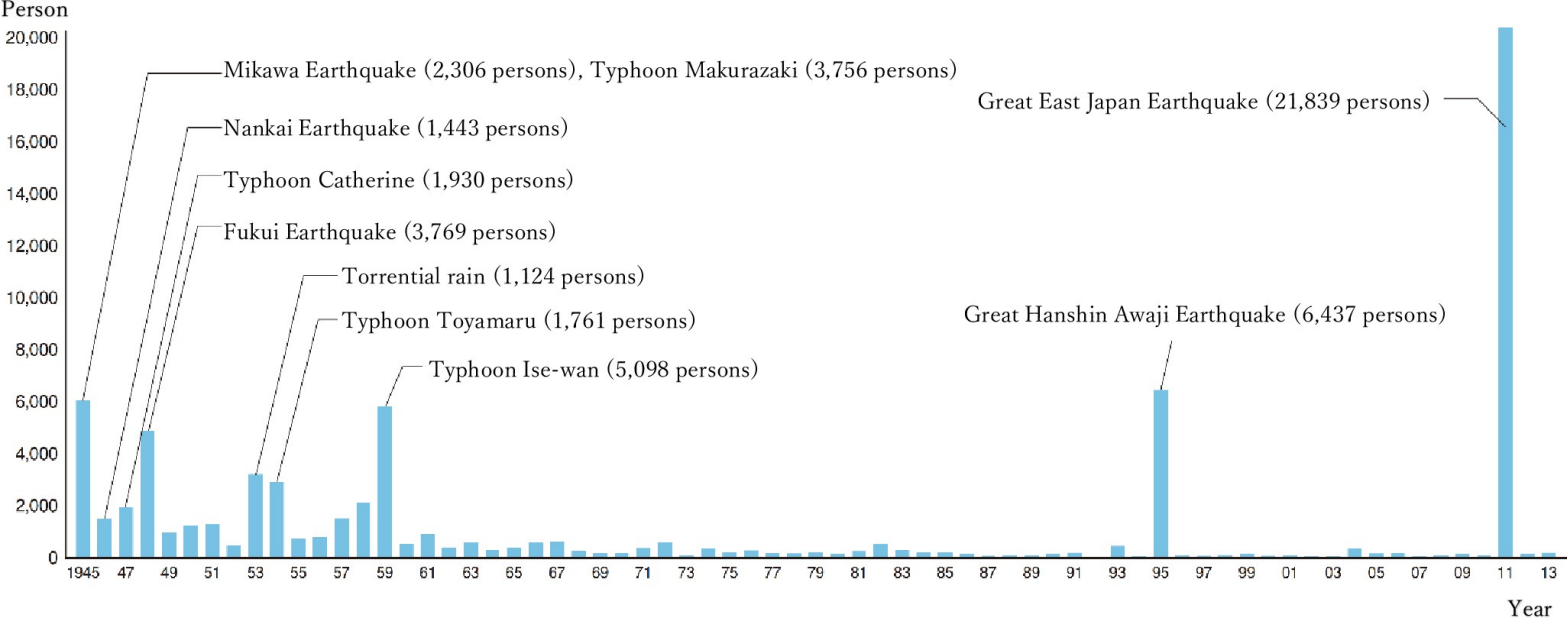
The number of deaths and missing persons caused by natural disasters. Source: Cabinet Office, Government of Japan “Disaster Management in Japan” Note: Representative disasters that occurred in each year are stated in the figure.

On March 11, 2011, the Great East Japan earthquake hit the Sanriku offshore. The Great East Japan earthquake, of which the magnitude was 9.0, is one of the most powerful earthquakes in Japanese history (see Fig 1). According to the Fire and Disaster Management Agency, as of April 1, 2018, 121,995 buildings were totally destroyed and 282,939 buildings were partially destroyed. Moreover, 19,689 people died with 2,563 missing.

According to police autopsy results, approximately 90% of the causes of death was drowning and 60% of the victims were old people. These figures indicate that the tsunami was the main channel through which damages were caused.

Such a tsunami experience can drastically change people’s evaluation of land. Because of the waterfront development boom, waterfront areas, of which elevations are low in general, were popular as residential and commercial areas during the 2000s. However, the experience of the Great East Japan earthquake has highlighted the risk of tsunamis in waterfront locations with low elevation. This paper aims to measure how the 2011 tsunami has changed people’s evaluation of land with different elevations and distances from the sea. In so doing, focus is placed on locations that are expected to be extensively damaged by a tsunami if the Nankai Trough earthquake occurs.

Our main findings are as follows. After the tsunami, locations with low elevation and close to the sea have experienced decreases in appraised land prices. Especially, locations with less than 3.6m elevation and within 1.46km of the coastline experienced significant decreases in appraised land prices. This implies that people have changed their location preferences.

The rest of this paper is organized as follows. Section 2 explains the research background of our study. Section 3 introduces data, and section 4 describes econometric specifications. Section 5 presents the results, and section 6 concludes.

## 2. Research background

Our study primarily relates to the literature of impacts of earthquakes and related disastars on land (property) prices [1]. showed the positive impact of the 1989 Loma Prieta earthquake on property prices in California. They explained that consumers initially overestimated the earthquake hazard, which results in the positive impacts [2–4]. showed the negative impacts of the Fukushima nuclear accident caused by the Great East Japan earthquake on the land prices [5]. revealed the negative impacts of the 2011 Oklahoma earthquake arising from the risk perception of wastewater injection [6]. examined how the experience of the Great East Japan earthquake changed housing rents and land prices of locations with different levels of risk by using hazard information. They showed that only locations that are supposed to be damaged most seriously (top 1~2%) have experienced decreases in housing rents and land prices. Compared to these studies, our study focuses on the impacts of tsunami risk that people recognize from the experience of the Great East Japan earthquake.

In this sense, our study is most closely related to [7] who empirically examined the impact of earthquake and tsunami’s risk on land prices. He focused on the Japanese government’s report on the possible damage of catastrophic earthquakes and tsunamis. He found that prefectures where the report was updated after the Great East Japan earthquake experienced decreases in land price. While he focused on the effects of risk information published by the government, we aim to uncover the effects of tsunami risk recognizable from location’s elevation and distance from the coastline, which have been overlooked in the literature.

Moreover, our study is possibly related to the literature of the flood impacts on property prices ([8–11]). Damages caused by tsunami partly include those caused by flood, which were examined in these studies. However, tsunami and flood are quite different. In the Great East Japan earthquake, the buildings located in areas hit by the tsunami were totally destroyed and over 10,000 people died directly from the tsunami damage, whereas flood damage is caused by rising water levels and the maximum number of flood victims is no more than 500 over the past half century in Japan. Put differently, tsunami damage cannot compare to flood damage in the current Japan. Moreover, tsunamis hit seaside areas whereas floods hit various areas. Hence, we believe that the tsunami risk changes people’s evaluation on land close to the sea more significantly than does flood risk, which is our focus.

## 3. Data

Data on appraised land prices comes from the Official Announcement of Land Prices published by the Ministry of Land, Infrastructure, Transport and Tourism (MLIT), Japan (see http://www.mlit.go.jp/en/totikensangyo/totikensangyo_fr4_000001.html). The Land Appraisal Committee of MLIT selects a location point in city planning areas (this is officially called a “standard site”), consults two or more real estate appraisers, and judges and publicly announces the proper land price per square meter once a year. It appraises the sites as of January 1 and announces prices in late March every year. A standard site is selected so that it can represent the average land usage in its neighborhood. The way of appraisal is determined by the Land Market Value Publication Act.

Land prices used in this paper are appraised by real estate appraisers and are not transaction prices. However, they are calculated primarily based on transaction prices of comparable lots in the neighborhood of the standard site and adjusted by using the capitalization method that summarizes possible (imputed) land rent revenues. MLIT specifies the detailed ways of calculation, which is built in the real-estate appraisal software supplied by companies approved by MLIT (e.g., https://www.tis.jp/service_solution/ko_kantei/, accessed on August 18, 2020). Although special treatments were applied to regions directly damaged by the earthquake and tsunami just after 2011, the ways of calculation have been unaltered for regions not directly damaged (see https://www.fudousan-kanteishi.or.jp/wp/wp-content/uploads/2017/06/24kouji_sinsai.pdf, accessed on August 18, 2020). Moreover, the appraised land price used in this paper is adjusted by normalizing site specific factors such as a lot shape, characteristics of the building on the lot (vacant, derelict, or newly-built etc.), characteristics of neighboring buildings. Hence, it can be thought of as the representative transaction price in the neighborhood of the standard site. Because our focus is not on site-specific factors, we use it rather than the transaction price. Moreover, althoughland transaction prices are available in the Land General Information System maintained by MLIT, it does not include information on the exact address of the transacted lot. Hence, we cannot obtain data on the lot’s elevation. Note further that the appraised land price doesn’t include the price of a building. Hence, it is not directly affected by building’s characteristics on the lot. Lastly, it is appraised once per year, implying its response to a particular shock is slower than that of the transaction price. Still, the earthquake and tsunami happened on March 2011, and the period of our focus is 2007–2016, which is sufficiently long for it to respond to the tsunami shock.

The Official Announcement of Land Prices reports not only appraised land price but also several land characteristics, which include acreage (*m*^2^), presence of supply facilities (supply of sewer and gas), the distance from the closest major traffic facilities (m), major regulations, floor area ratio, etc. Here, the major traffic facilities include train stations and bus stops (bus stations). We’ve found that non-negligible share (around 13%) of standard sites experienced changes in the distance from the closest major traffic facilities during our sample periods. One of the major reasons for this is the new construction or closure of bus stops. For land with any buildings, the Official Announcement of Land Prices also reports building characteristics, which include building coverage, number of floors above ground, etc. As stated, such building characteristics do not directly affect the land price. However, some of them reflect the complicated regulations on land usage, which would affect land price. Hence, we include such building characteristics in our regression. Table 1 shows the descriptive statistics.

**Table 1:** Descriptive statistics.

	Variables	Observations	Mean	Std. Dev.	Min	Max
*Standard site’s characteristics*						
	Logarithm of appraised land price per *m*^2^	11,624	10.9139	0.6960109	0	14.31021
	Elevation (m)	11,624	36.18896	74.05576	-1.2	720.4
	Distance from the coastline (m)	11,624	6671.748	9304.083	19.93617	67752.79
	Acreage of land (*m*^2^)	11,624	413.233	2674.804	49	100000
	Distance from the closest major traffic facilities (m)	11,624	2407.694	3374.982	0	38000
	Number of floor above ground	11,624	2.127753	0.9108638	1	10
	Building coverage ratio (%)	11,624	63.66225	9.271856	0	80
	Floor area ratio (%)	11,624	215.7252	92.96334	0	600
*Regulation dummy*						
	Residential area	11,624	0.6693909	0.4704527	0	1
	Commercial area	11,624	0.2437199	0.4293441	0	1
	Industrial area	11,624	0.0127323	0.1121216	0	1
	Quai-industrial area	11,624	0.0205609	0.1419151	0	1
*Supply facility dummy*						
	Gus	11,624	0.4650723	0.4988	0	1
	Sewer	11,624	0.6233655	0.4845629	0	1

The land sizes vary widely (from 49 to 100,000 square meters) in our sample. However, as shown in–A1 in S1 Appendix, the land size distribution of the treatment group looks very similar to that of the control group. Because the DID (DDD) approach estimates the average difference between the treatment and control groups after the event, our estimation results are not biased due to differences in land sizes.

A few comments are in order. First, Japan’s City Planning Act regulates the possible land and property usage in a particular area, and defines "Land Use Zones." It is established to support efficient urban activities, achieve a pleasant urban environment, and create townscapes with significant features. Land Use Zones consists of twelve types of zones, and are often categorized as residential areas, commercial areas and industrial areas (see https://www.mlit.go.jp/common/001050453.pdf). In our data, which is published by the MLIT, these twelve types of zones are categorized into four: residential areas, commercial areas, industrial areas, and quasi-industrial areas. A residential area is used as a site for constructing residences, and contains seven types of zones. A commercial area is used as a site for constructing commercial facilities, and has two types of zones. An industrial area is used as a site for constructing factories, and has two types of zones. A quasi-industrial area is used as a site for constructing factories of light industries excluding environmental degradation plants and highly hazardous plants: it has one type of zones. However, if the actual land use differs from the land use designated by Land Use Zones, the MLIT reports the former instead of the latter. Second, our variable “Building coverage ratio” represents the ratio of building area to site area. The Building Standards Act sets limits on it to prevent a fire for each region and land use. Finally, changes caused by the tsunami would be more prominent for land wherein people reside. Hence, focus is placed only on land with buildings.

We include commercial, quasi-industrial, and industrial areas in our analysis. This is because we want to capture the overall tsunami impacts on land prices. In fact, some of the local governments and private companies have moved their offices or warehouses to locations far from the coastline or with high elevation. We want to capture their impacts as well as households’ side impacts. However, some existing works focus only on residential areas. For the sake of comparison, we conducted the estimation (2) by using only the residential area data. Table A3 in S1 Appendix shows the results, which are mostly the same as our main results shown in Table 4.

Next, the data on elevation and distance from the coastline is matched to the data on appraised land prices. The data on elevation comes from the Geospatial Information Library (MLIT). The data on distance from the coastline is computed by using the Geographic Information System software, ArcGIS.

Since focus is on the changes in land evaluation through the tsunami experience and not on the changes caused by direct damages, focus is on the western Japan. Specifically, focus is on six prefectures: Miyazaki, Kochi, Tokushima, Mie, Wakayama, and Shizuoka. This is because that the Nankai Trough earthquake is one of the largest possible earthquakes that might occur in the future, and these six prefectures are predicted to be damaged from it most extensively. In fact, the Headquarters for Earthquake Research Promotion (Ministry of Education, Culture, Sports, Science, and Technology, Japan) reports its probability within 30 years as 0.7–0.8 (https://www.jishin.go.jp/regional_seismicity/rs_kaiko/k_nankai/, accessed on September 5, 2019).

According to the Central Disaster Management Council, the maximum death toll could be as many as 323,000, of which death by tsunami would amount to 230,000. Maximum possible economic loss could be approximately 170 trillion yen for assets and 45 trillion yen for degradation of production and services.

**Fig 2:**
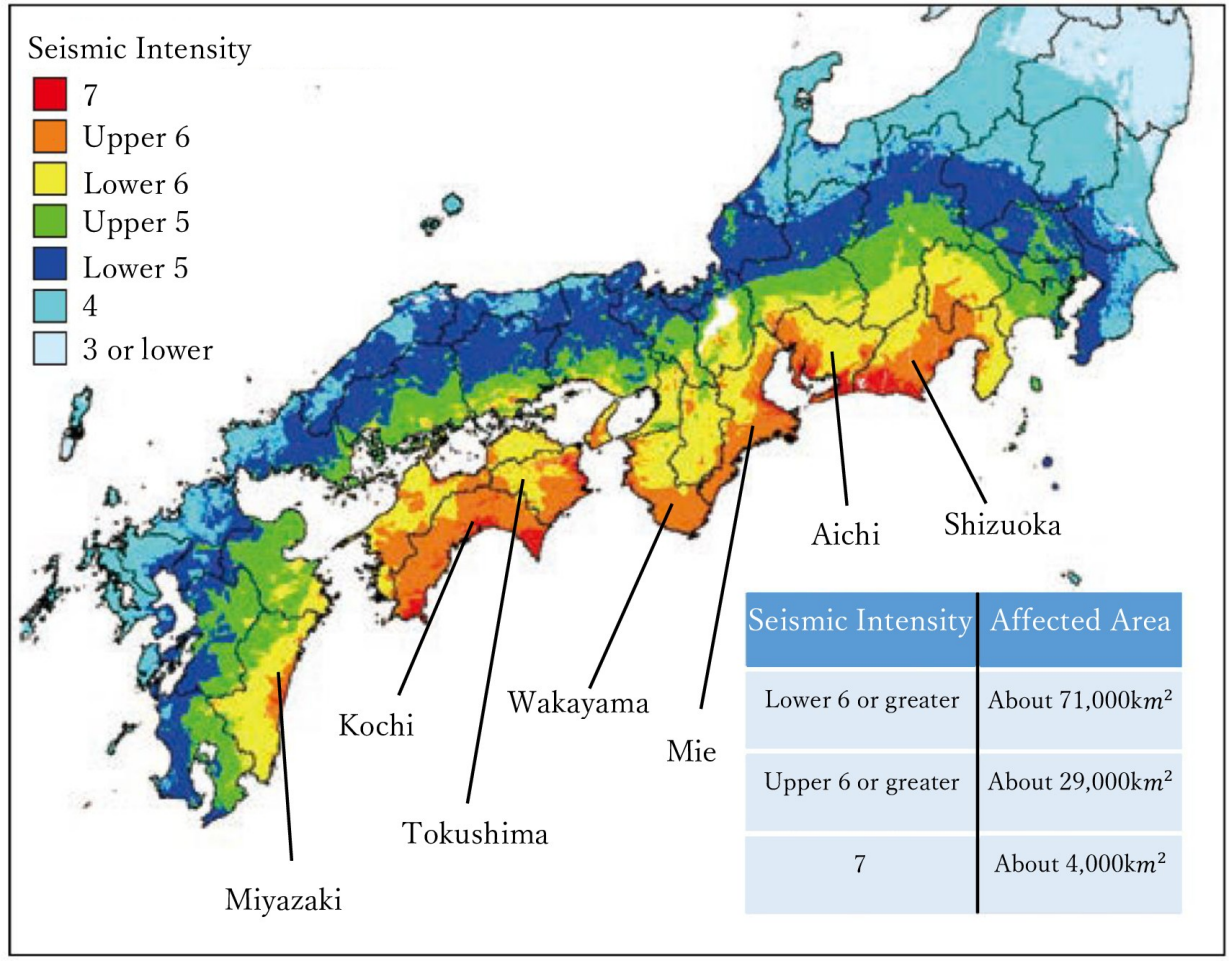
Distribution of maximum seismic intensity (shindo) in the event of a maximum possible earthquake. Source: Cabinet Office, Government of Japan “Disaster Management in Japan”.

**Fig 3:**
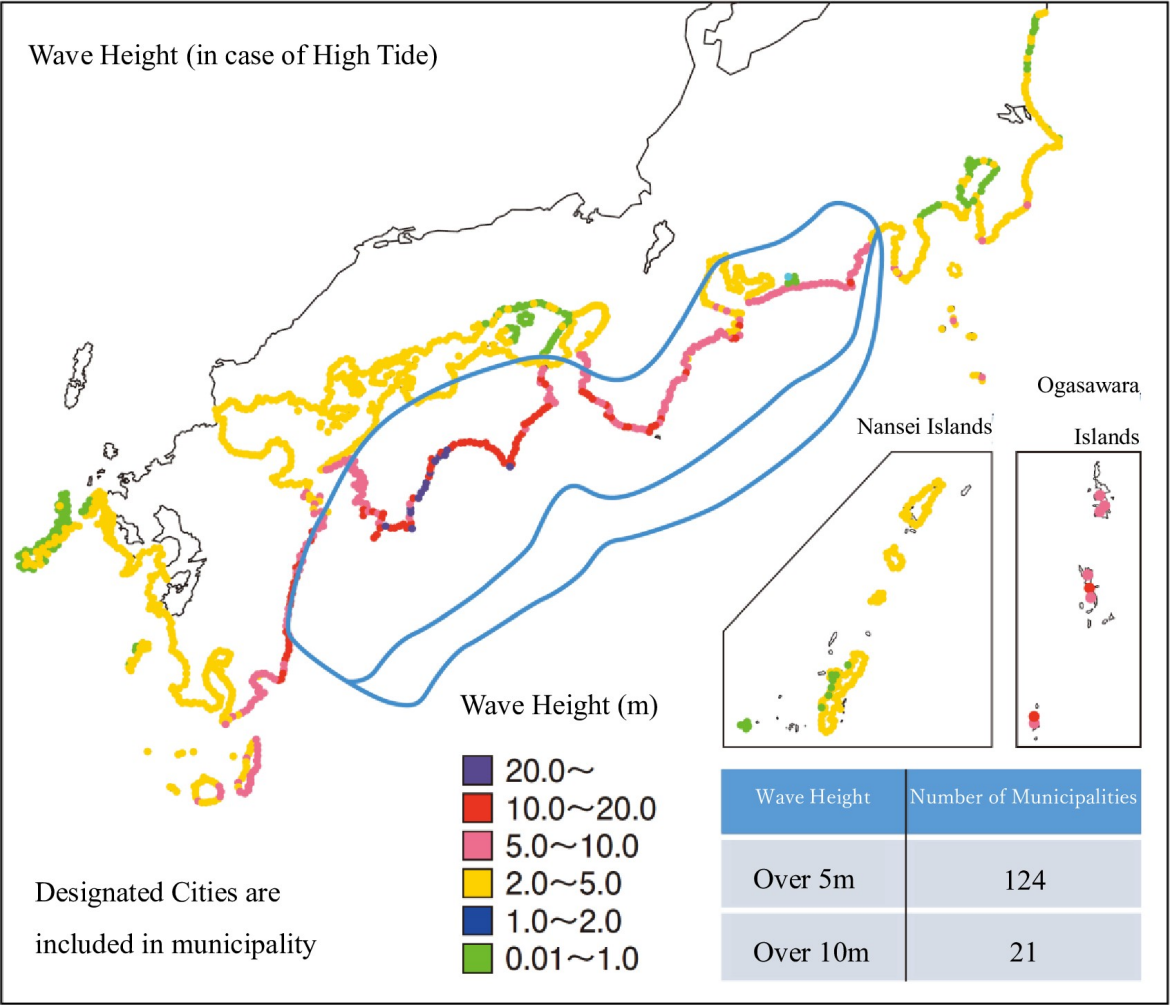
Distribution of tsunami wave height in the event of a maximum possible earthquake. Source: Cabinet Office, Government of Japan “Disaster Management in Japan” Note: The government estimated the earthquake damage in every conceivable situation. This diagram represents one of them.

If the earthquake occurs, Fig 2 shows the distribution of maximum seismic intensity (Shindo) and Fig 3 shows the distribution of tsunami wave height. The shown prefectures are predicted to be extensively damaged (http://www.mlit.go.jp/river/earthquake/en/nankai/index.htm).

Hence, we expect that the tsunami experience caused by the Great East Japan earthquake has significantly changed land evaluation of people residing in these prefectures. Our sample period covers from 2007 to 2016, where the Great East Japan earthquake occurred in 2011. We have 1,166 standard sites in the above six prefectures. Each year MLIT replaces some of the standard sites with new ones in order to ensure that a standard site can represent the average land usage in its neighborhood and so the sample size becomes 11,624, which is somewhat smaller than 1,166×10.

The dataset does not contain the points of Aichi prefecture, which are also predicted to be extensively damaged by tsunami. This is because the land price trend in Aichi prefecture differs from the other six prefectures. Figs A2 and A3 in S1 Appendix indicate that trends of average land prices for locations in prefectures that are expected to be affected by the tsunami. The trends of land price in the six prefectures are downward sloping; however, in the Aichi prefecture it is slightly U-shaped and has remained higher in price than other prefectures. Moreover, Aichi prefecture conducted several large-scale re-development projects during the 2000’s. Especially, one of them re-developed the waterfront areas during the 2010’s, and a large-scale commercial center called Maker’s Pier (https://www.makerspier.com/en/) opened in 2015. Because other prefectures have not experienced such re-development projects in the waterfront areas, we remove the sample in Aichi prefecture from our main analysis (We also conduct estimation by including Aichi prefecture, and confirm that our main results are unaltered. See Table A4 in S1 Appendix).

## 4. Econometric specifications

This paper uses the difference-in-differences approach to uncover the impact of the tsunami. Here, we focus on locational differences in elevation and distance from the sea, and estimate two specifications. The first specification examines the impact on locations with different elevation or distance from the coastline. The second one includes a cross-term of the elevation and distance variables. This is because we expect the effects of the tsunami on appraised land prices to differ for locations with different distances from the coastline, being significant for locations close to the sea while non-significant for distant locations.

**Fig 4:**
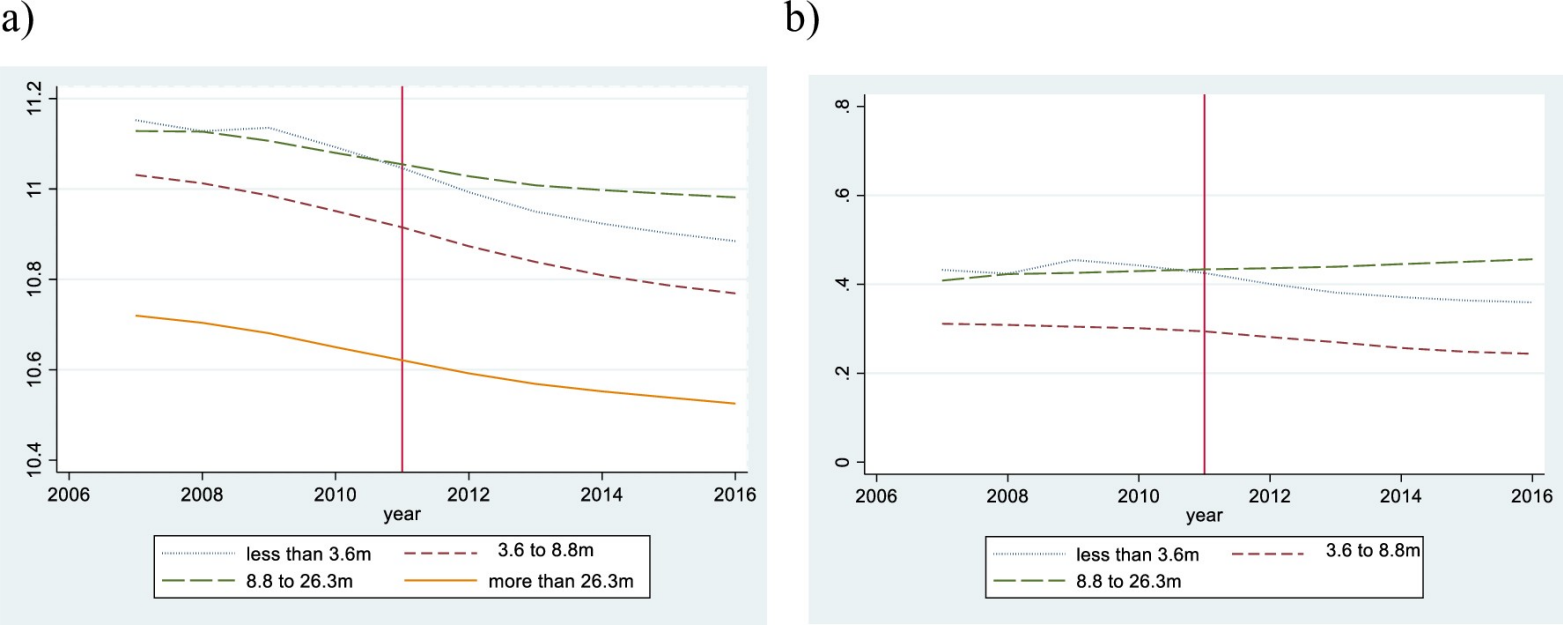
The average appraised land price by elevation. Note: Fig 4A shows the average appraised land price by elevation, and Fig 4B shows the difference between the treatment group and the control group by elevation.

**Fig 5:**
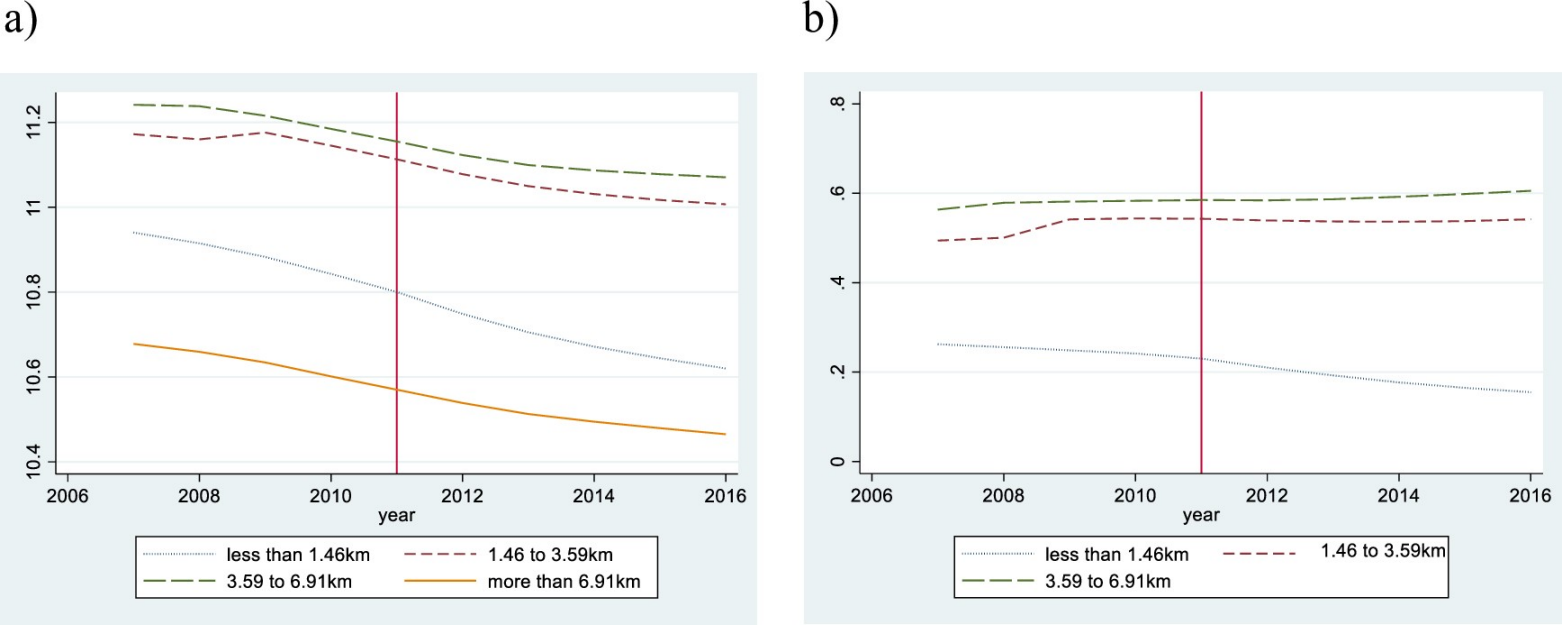
The average appraised land price by distance from the coastline. Note: Fig 5A shows the average appraised land price by distance from the coastline, and Fig 5B shows the difference between the treatment group and the control group by distance from the coastline.

In both estimations, the dependent variable is ln(*p*_*it*_), which denotes the logarithm of appraised land price per square meter of site i at time t. The after-treatment dummy is denoted by *after*_*t*_, site i’s attribute vector by ***X***_***it***_, and the linear time trend by *Trend*_*t*_. As shown in Figs 4A and 5A, we found that the time trend of land price exhibits slight non-linearity. Hence, we added a square term to represent the trend appropriately. ***X***_***it***_ consists of i’s land and building characteristics. It includes the acreage of the land (*m*^2^), the dummy variables of presence of supply facilities (supply of sewer, and gas), the distance from the closest major traffic facilities (m), the regulation dummy, the building coverage ratio, the floor area ratio, and the number of floor above ground. The regulation dummy controls for the effects of zoning, which categorizes locations into the following 5 areas: the urbanization control area, residential area, commercial area, industry area, and quasi-industrial area. To control for time-invariant land and building characteristics, we include the site i fixed effect.

In our regression, we include the site fixed effect, “*FE*_*i*_” in addition to site characteristics. Note here that i represents a site. Hence, if a particular land regulation changes or an infrastructure facility is renovated, site i’s characteristics can change. In fact, we confirm that some of the characteristics (e.g., “floor area ratio”, “building coverage ratio”, and “regulations”) have changed during our sample term (ten years). Hence, we use the characteristics to capture observable time-variant effects, and “*FE*_*i*_” to capture unobservable time-invariant effects. We try to remove the omitted variables bias by including the site fixed effect (*FE*_*i*_) and some building characteristics such as the height of buildings. The former is included to control the effect of time-invariant variables such as proximity to business center, proximity to historic district, and historic nature etc. For the latter, we use building characteristics reflecting the complicated regulations on land usage.

We tested for independence and homoscedasticity of the error term for estimation (2) in Table 4, and found that the error term is serially correlated, cross-sectionally dependent, and heteroscedastic (The test results are available upon request). Therefore, we use the [12]’ s standard error. We thank the reviewer for pointing this out.

In the first specification, we estimate the impacts of the tsunami caused on appraised land prices of locations with different elevation or distance from the coastline. We specify the estimation equation as

$${\ln p}_{it}=\alpha +\sum_{j}\beta_{1j}(aft{er}_{t}\times I_{lji})+\beta_{2}\times {after}_{t}+X_{it}\beta_{3}+{Trend}_{t}+{Trend}_{t}^{2}+{FE}_{i}+e_{it}$$ (1)

*I*_*lji*_ is the dummy variable for elevation or distance from the coastline at site *i*. Here, *l*∈{A, D} where A represents elevation and D represents the distance from the coastline. *j* represents a category for each *l*. *I*_*Aji*_ is the dummy variable representing elevation, and the sites are categorized by the following criteria: under 3.6m / 3.6m to 8.8m / 8.8m to 26.3m. We categorized them to keep each group’s sample size similar (approximately 3,000 in each group). ${I_{D}}_{ji}$ is the dummy variable representing distance from the coastline, and the sites are categorized by the following criteria: less than 1.46km / 1.46km to 3.58km / 3.58km to 6.91km. Again, samples are categorized to keep each group’s sample size similar. *α* is a constant and *e*_*it*_ is the error term. βs are estimated coefficients and a bold character represents a vector. Hence, the control group in (1) consists of sites with elevation higher than 26.3m if we use *I*_*Aji*_ and sites more than 6.91km further away from the coastline if we use ${I_{D}}_{i}$.

Next, we confirm the parallel trend of appraised land prices before the tsunami. Fig 4A shows the average appraised land price by elevation. Before the tsunami, their time-trends draw a downward-sloping curve, regardless of elevation. Fig 4B shows the difference between the treatment group and the control group by elevation. The appraised land price trends of the treatment groups hardly differed from those of the control group before the tsunami. Fig 5A shows the average appraised land price by distance from the coastline and their time-trends draw a downward-sloping curve. Fig 5B shows the difference between the treatment group and the control group by distance from the coastline, and the appraised land price trends of the treatment groups hardly differed from those of the control group before the tsunami. Hence, we confirm that our treatment and control groups satisfy the parallel trend assumption.

The coefficient of our interest is *β*_1*j*_. If dummies are used for elevation, i.e., *I*_*Aji*_, it captures differences in the pre- to post-disaster changes in appraised land prices of locations with different elevations compared to the control group. If dummies are used for distance from the coastline, i.e., *I*_*Dji*_, it captures differences in the pre- to post-disaster change in appraised land prices of locations with different distances from the coastline compared to the control group.

In the first specification, the impact of the tsunami on appraised land prices of locations with different elevations and different distances from the coastline are estimated independently. However, the possible damages caused by the tsunami would be extremely large at locations with low elevation and close to the coastline, and they would be moderate at locations with low elevation but distant from the coastline or those close to the coastline but with high elevation. To investigate this possibility, in the second specification, we add a cross-term of elevation and distance dummies, called DDD (Difference-in-Difference-in-Difference) estimation method.

$${\ln p}_{it}=\alpha +\sum_{k}\sum_{j}\beta_{1jk}(after_{t}\times I_{Aji}\times I_{Dki})+\sum_{j}\beta_{2j}({after}_{t}\times I_{Aji})+\sum_{j}\beta_{3j}({after}_{t}\times I_{Dji})+\beta_{4}\times {after}_{t}+X_{it}\beta_{5}+{Trend}_{t}+{Trend}_{t}^{2}+{FE}_{i}+e_{it}$$ (2)

Hence, the control group in (2) consists of sites with elevations higher than 26.3m and more than 6.91km further away from the coastline. We used the same grouping criteria regarding *I*_*lji*_ as (1). The coefficient of interest variable is *β*_1*jk*_. We estimate *β*_1*jk*_ for each classified group, depending on the elevation and the distance from the coastline, so that there are 9(3×3) treatment groups.

## 5. Results

Table 2 shows the result of estimating (1) when focus is on elevation. The treatment group, which consists of locations with elevations lower than 26.3m, is divided into 3 subgroups to focus on differences in the tsunami’s effects.

**Table 2:** DID estimation result: Elevation and appraised land price.

	(1)
Variables	DID
After	-0.00692
	(0.00641)
After × elevation less than 3.6 m	-0.0645***
	(0.0108)
After × elevation 3.6 to 8.8 m	-0.0477***
	(0.0106)
After × elevation 8.8 to 26.3 m	0.0223**
	(0.00755)
Constant	12.25***
	(0.666)
Standard site’s FE	Yes
Standard site’s characteristics	Yes
Building’s characteristics	Yes
Trend	Yes
Observations	11,624
Number of standard sites	1,166
R-squared	0.234

Note

***, **, and * indicate that the estimated coefficient is significant at the 0.01, 0.05, and 0.10 levels, respectively. [12]’s standard error are reported in parentheses. Standard site’s characteristics are as follows: acreage of the land (*m*^2^), distance from the major traffic facilities (m), dummy variables of presence of supply facilities (supply of sewer, and gas), dummy of regulation (residential area, commercial area, industrial area, quasi-industrial area), building coverage ratio, floor area ratio, and number of floor above ground. We also control standard site’s fixed effect, and linear time trend but omit them from the result.

In Table 2, the estimated *β*_1*j*_s are negative and significant for sites with low elevation (less than 8.8m). In contrast, *β*_1*j*_s are positive and significant for locations with relatively high elevation (8.8 to 26.3m). Thus, we can confirm that people have decreased their evaluation of locations with low elevation and increased their evaluations of locations with relatively high elevation after the tsunami.

Next, Table 3 shows the result of estimating (1) when focusing on distance from the coastline. The treatment group, which consists of locations with distances from the coastline lower than 6.91km, is divided into three subgroups to focus on differences in the tsunami’s effects.

**Table 3:** DID estimation result: Distance from the coastline and appraised land price.

	(1)
Variables	DID
After	-0.0185*
	(0.00894)
After × distance less than 1.46 km	-0.0710***
	(0.0156)
After × distance 1.46 to 3.58 km	0.0117
	(0.0118)
After × distance 3.58 to 6.91 km	0.0166**
	(0.00667)
Constant	12.31***
	(0.638)
Standard site’s FE	Yes
Standard site’s characteristics	Yes
Building’s characteristics	Yes
Trend	Yes
Observations	11,624
Number of standard sites	1,166
R-squared	0.234

Note

***, **, and * indicate that the estimated coefficient is significant at the 0.01, 0.05, and 0.10 levels, respectively. [12]’s standard error are reported in parentheses. The set of explanatory variables used in the model is identical to that of Table 2. We also control standard site’s fixed effect, and linear time trend but omit them from the result.

In Table 3, the estimated *β*_1*j*_s are negative and significant for locations with distance from 0 to 1.46km. Although *β*_1*j*_s are positive for locations with distance from 1.46 to 6.91km, they are non-significant. Thus, we can confirm that people have decreased their evaluation of locations very close to the sea after the tsunami.

Finally, the result of estimating (2) is presented in Table 4, which reports the estimated *β*_1*jk*_. In the DDD regression, the treatment group is divided into 9 (3 × 3) subgroups by elevation and distance from the coastline. Thus, the control group in this estimation consists of locations with elevation equal to 26.3 m or more and distance from the coastline equal to 6.91km or more.

**Table 4:** DDD estimation result.

Distance from the coastline				
3.58 km to 6.91 km	-0.0901***	-0.0426**	-0.0103**	
	(0.0233)	(0.0169)	(0.00405)	
1.46 km to 3.58 km	-0.0565***	0.0297*	0.00617	
	(0.0171)	(0.0155)	(0.00421)	
within 1.46 km	-0.109***	-0.0516	-0.0941**	
	(0.0320)	(0.0303)	(0.0304)	
	less than 3.6m	3.6 to 8.8 m	8.8 to 26.3 m	
				Elevation

Note: The table shows the estimated *β*_1*jk*_ in Eq (2).

***, **, and * indicate that the estimated coefficient is significant at the 0.01, 0.05, and 0.10 levels, respectively. [12]’s standard error are reported in parentheses. The set of explanatory variables used in the model is identical to that of Table 2. We control after-treatment dummy, land fixed effect, and linear time trend. Moreover, we estimate the coefficients *β*_2*j*_ and *β*_3*j*_ in Eq (2). But they are omitted from the result.

In Table 4, the estimated *β*_1*jk*_s are mostly negative. The locations with low elevation (under 3.6m) and close to the coastline (0 to 1.46km) have experienced significant and large declines in appraised land price. In addition, we can see that the estimated *β*_1*jk*_s are also negative though small in absolute value even for locations somewhat distant from the sea (3.58 to 6.91km) and for locations with relatively high elevation (8.8 to 26.3m) if the location is close to the sea, i.e., 0 to 1.46km. These imply that such locations experienced slight declines in appraised land prices compared with locations with even higher elevation and further distant from the sea. Moreover, we observe increases in appraised land prices for locations with moderate elevation (3.6 to 8.8m) and distanec from the sea (1.46 to 3.58km). This would reflect changes in people’s preference from locations with very low elevation and very close to the sea to these locations. Thus, the results of estimating (1) and (2) imply that people have come to avoid locations with low elevation and close to the sea. In the S1 Appendix, we try two placebo tests. First, we estimate (2) by using a different sample that consists of prefectures predicted to bear no damage by a tsunami if the Nankai Trough earthquake occurs. Second, we estimate (2) by using a different sample from 2007 to 2011, where we will expect no effects of the tsunami. In both placebo tests, we do not find clear-cut changes in people’s preference as shown in Table 4. Hence, it is confirmed that the treatment effects in Table 4 capture the effects of the tsunami caused by the Great East Japan earthquake. Our estimation results can potentially include the effect of seismic risk because tsunamis are caused by earthquakes. However, we believe that the impact of earthquakes on land prices is quite small in our analysis. This is mainly because damage directly caused by earthquakes has been relatively small in recent Japan. The Japan Meteorological Agency has recorded damages caused by huge earthquakes, which have seismic intensity 4 or greater and magnitude 5 or greater (https://www.data.jma.go.jp/svd/eqev/data/higai/higai1996-new.html). Since 2000, in most cases without tsunamis, the number of dead and totally destroyed buildings are both zero. In fact, according to the police autopsy results, in the Great East Japan earthquake, approximately 90% of the causes of death were drowning, which is mostly caused by the tsunami. Therefore, we consider that our estimation results come mostly from the impact of tsunami risk.

## 6. Concluding remarks

This paper estimates the impact of the tsunami caused by the Great East Japan earthquake on land appraisals of various locations outside of directly damaged areas. Focus is placed on locations that are supposed to be extensively damaged by a tsunami if the Nankai Trough earthquake occurs. Using the DID and DDD approaches, the impact differences between before and after the tsunami from the Great East Japan earthquake are observed.

Focus is on the elevation and distance from the coastline. As a result, we find that locations with low elevation and close to the sea experienced decreases in appraised land prices compared to locations with high elevation and far from the coastline after the tsunami. People have changed their preference for location regarding elevation and distance from the sea after experiencing the tsunami.

Note here that we base our analysis on land appraisals. As we explained, they are calculated primarily based on transaction prices. Hence, we believe that they reflect risk perceptions of agents to some extent. However, they also include other adjustments by appraises, which might cause some bias. We must keep this limitation in our mind.

Finally, in the future, we would like to use microdata on people’s relocation to examine whether people actually move from locations experiencing decreases in appraised land prices to locations experiencing increases in appraised land prices.

## Supporting information

S1 Appendix (DOCX)

S1 Table Estimation result of all control variables in Table 2. DID Estimation Result: Elevation and Appraised Land Price.
(DOCX)

S2 Table Estimation result of all control variables in Table 3. DID Estimation Result: Distance from the Coastline and Appraised Land Price.
(DOCX)

S3 Table Estimation result of all control variables in Table 4. DDD Estimation Result.
(DOCX)

S4 Table Estimation result of all control variables in Table A1. DDD Estimation Results Western Japan Sample without Tsunami Risk.
(DOCX)

S5 Table Estimation result of all control variables in Table A2. DDD Estimation Results with a Different Sample Period.
(DOCX)

S6 Table Estimation result of all control variables in Table A3. DDD Estimation results for the Residential Areas.
(DOCX)

S7 Table Estimation result of all control variables in Table A4. DDD Estimation Results by Including Aichi Prefecture.
(DOCX)
