# Supplementary material for: The impact of Tsunamis on land appraisals: Evidence from Western Japan
Source: PLoS One. 2021 Apr 6;16(4):e0248860. doi: 10.1371/journal.pone.0248860 (PMC8023538; doi:10.1371/journal.pone.0248860)
Supplement: S1 Appendix — (DOCX) [file pone.0248860.s001.docx]

**S1 Appendix.**

# Appendix A: Placebo Tests

The result in section 4 implies that people have changed their preference for location from relatively low locations to high locations triggered by the tsunami experience. We try two placebo tests.

First, we change the sample from areas predected to be damaged by by a tsunami if the Nankai Trough earthquake occurs to areas predected not to be damaged by it.

If the changes in appraised land prices in these areas are the same as those in Table 4, the changes shown in Table 4 do not necessarily capture the actions taken to avoid tsunami damage if the Nankai Trough earthquake occurs. However, if not, the changes in appraised land prices shown in Table 4 imply actions taken to avoid tsunami damage in prefectures that face the risk of a tsunami.

Then, we estimate (2) as in Table 4, but, the sample is changed to western Japan excluding Achi, Kochi, Mie, Miyazaki, Shizuoka, Tokushima, and Wakayama prefectures. We have 5,189 sites in these 18 prefectures, implying that our sample size is 51,767. Table A1 in S1 Appendix shows the estimation result. While we observed decreases in appraised land prices for locations with high elevation (8.8 to 26.3m), we found no statistically significant effect for locations with low elevation and close to the coastline (0 to 1.46km). Therefore, the change in appraised land price shown in Table 4 is found to be an action to avoid the tsunami damage.

**Table A1. DDD Estimation Results for Western Japan Sample without Tsuanmi Risk.**

| Distance from the coast line | |  |  |  |  |
| --- | --- | --- | --- | --- | --- |
|  | 3.58 km to 6.91 km | -0.00658 | 0.0167*** | -0.0272*** |  |
|  |  | (0.0129) | (0.00465) | (0.00794) |  |
|  | 1.46 km to 3.58 km | -0.0274* | -0.0360*** | -0.0304*** |  |
|  |  | (0.0122) | (0.00760) | (0.00643) |  |
|  | within 1.46 km | 0.0119 | -0.00495 | -0.0137*** |  |
|  |  | (0.00822) | (0.00440) | (0.00411) |  |
|  |  | less than 3.6m | 3.6 to 8.8 m | 8.8 to 26.3 m |  |
|  |  |  |  |  | Elevation |

Note: The table shows the estimated $\beta_{1jk}$ in equation (2). ***, **, and * indicate that the estimated coefficient is significant at the 0.01, 0.05, and 0.10 levels, respectively. [12]’s standard error are reported in parentheses. The set of explanatory variables used in the model is identical to that of Table 4. We control after-treatment dummy, land fixed effect, and linear time trend. Moreover, we estimate the coefficients $\beta_{2j}$ and $\beta_{3j}$ in equation (2). But they are omitted from the result.

Second, we conduct the DDD estimation as if the shock occurred at the end of 2009. Hence, we define “before” from 2007 to 2009 and “after” from 2010 to 2011. We focus on the difference in appraised land price between treatment group and control group before the tsunami, to confirm the change of appraised land price shown in Table 4 is affected by the tsunami on March 11, 2011.

If the changes in appraised land prices between treatment group and control group before the tsunami experience are the same as those in Table 4, then the difference in appraised land price between the treatment group and control group have existed before the tsunami experience. In such a case, the difference shown in Table 4 does not necessarily capture the effect of the tsunami experience caused by the Great East Japan earthquake. However, if not, there is no difference between the treatment group and control group before the tsunami experience and the changes in appraised land prices shown in Table 4 imply the effects of the tsunami.

Then, we use DDD estimation method (2) as in Table 4. We have 1,166 sites and our sample size becomes 5,813. Table A2 in S1 Appendix shows the estimation result. The estimated $\beta_{1jk}$s exhibit little clear-cut patterns, which are in contrast to those shown in Table 4. Moreover, the values of significantly estimated coefficients in Table A2 in S1 Appendix for locations with low elevation (less than 3.6m) or close to the coastline (0 to 1.46km) are -0.03 to -0.04, of which magnitude is much smaller than that of the corresponding figures in Table 4. It implies that most of the changes in appraised land prices shown in Table 4 capture the effects of the tsunami experience.

**Table A2. DDD Estimation Results with a Different Sample Period.**

| Distance from the coast line | |  |  |  |  |
| --- | --- | --- | --- | --- | --- |
|  | 3.58 km to 6.91 km | -0.0434** | 0.00295* | 0.00550* |  |
|  |  | (0.0105) | (0.00116) | (0.00249) |  |
|  | 1.46 km to 3.58 km | 0.0103 | 0.0388** | 0.0155** |  |
|  |  | (0.0134) | (0.0107) | (0.00392) |  |
|  | within 1.46 km | -0.0347** | 0.0214*** | -0.0311** |  |
|  |  | (0.00771) | (0.00449) | (0.00909) |  |
|  |  | less than 3.6m | 3.6 to 8.8 m | 8.8 to 26.3 m |  |
|  |  |  |  |  | Elevation |

Note: The table shows the estimated $\beta_{1jk}$ in equation (2). ***, **, and * indicate that the estimated coefficient is significant at the 0.01, 0.05, and 0.10 levels, respectively. [12]’s standard error are reported in parentheses. The set of explanatory variables used in the model is identical to that of Table 4 except for regulation dummies. We control after-treatment dummy, land fixed effect, and linear time trend. Moreover, we estimate the coefficients $\beta_{2j}$ and $\beta_{3j}$ in equation (2). But they are omitted from the result.

# Appendix B: Other Tables and Figures

**Fig A1. The Land Size Distribution between the Treatment and Control Groups.** Note: In this figure, the control goup is same as (2), which consists of sites with elevations higher than 26.3m and more than 6.91km further away from the coastline, and the treatment group consists of other sites.


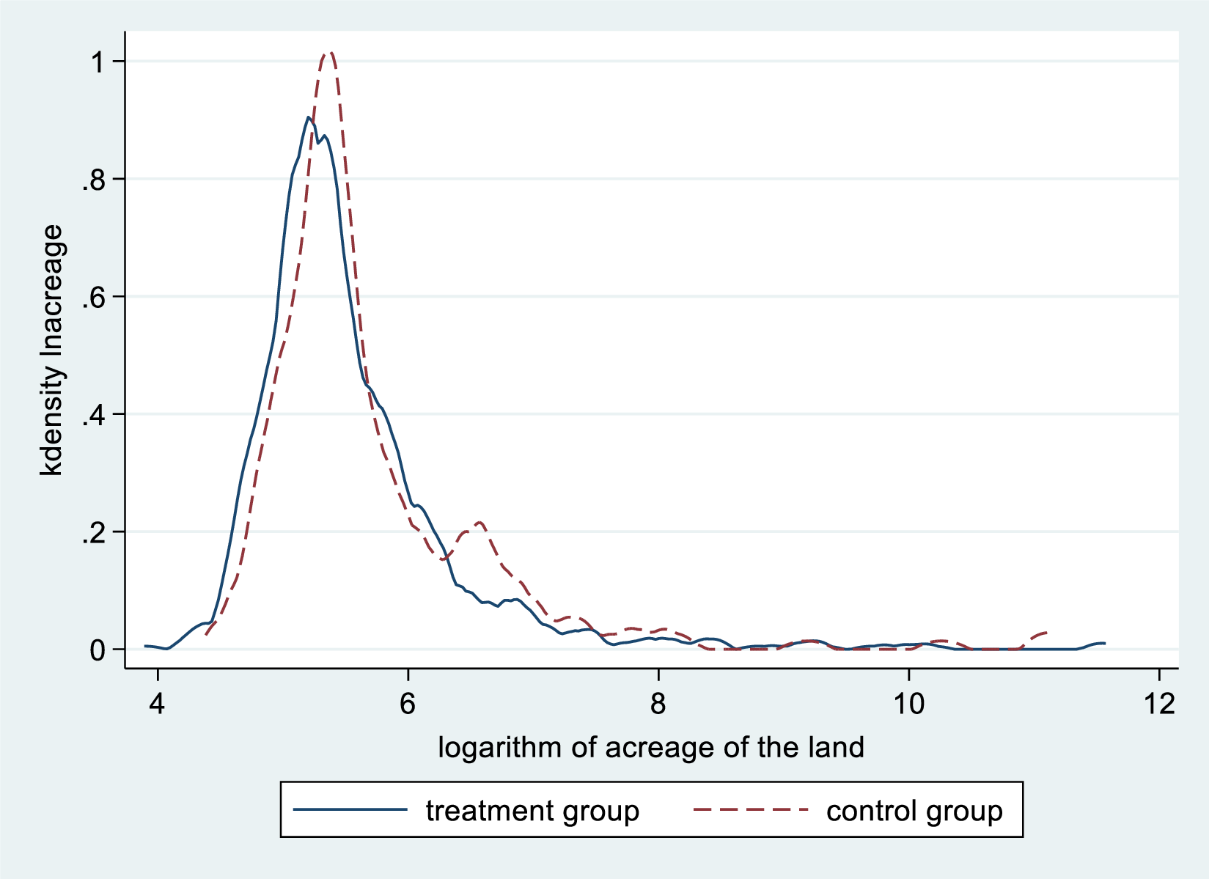


**Fig A2. The Average Appraised Land Price of Locations with Elevations Higher than 26.3m by Prefecture.**

**
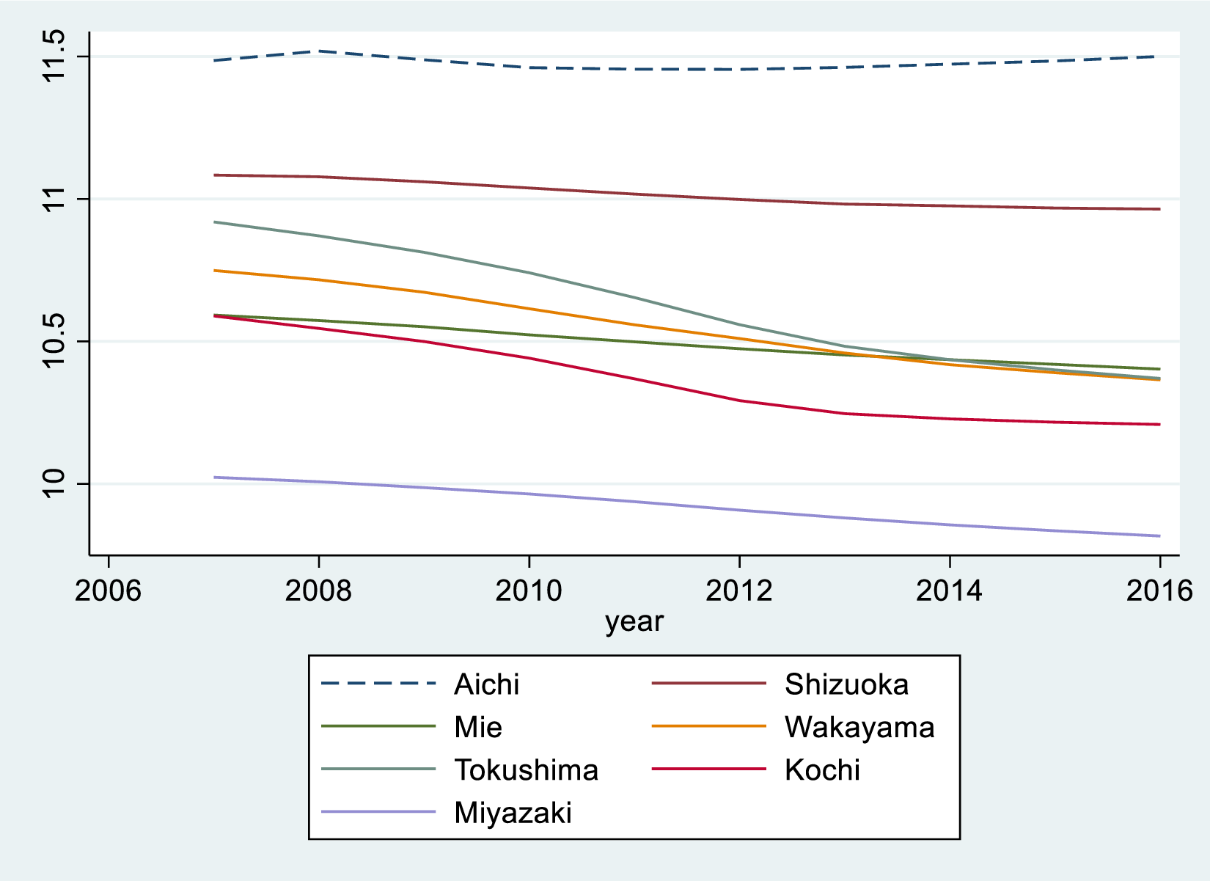
**

**Fig A3. The Average Appraised Land Price of Location over 6.91km away from the Coastline by Prefecture.**


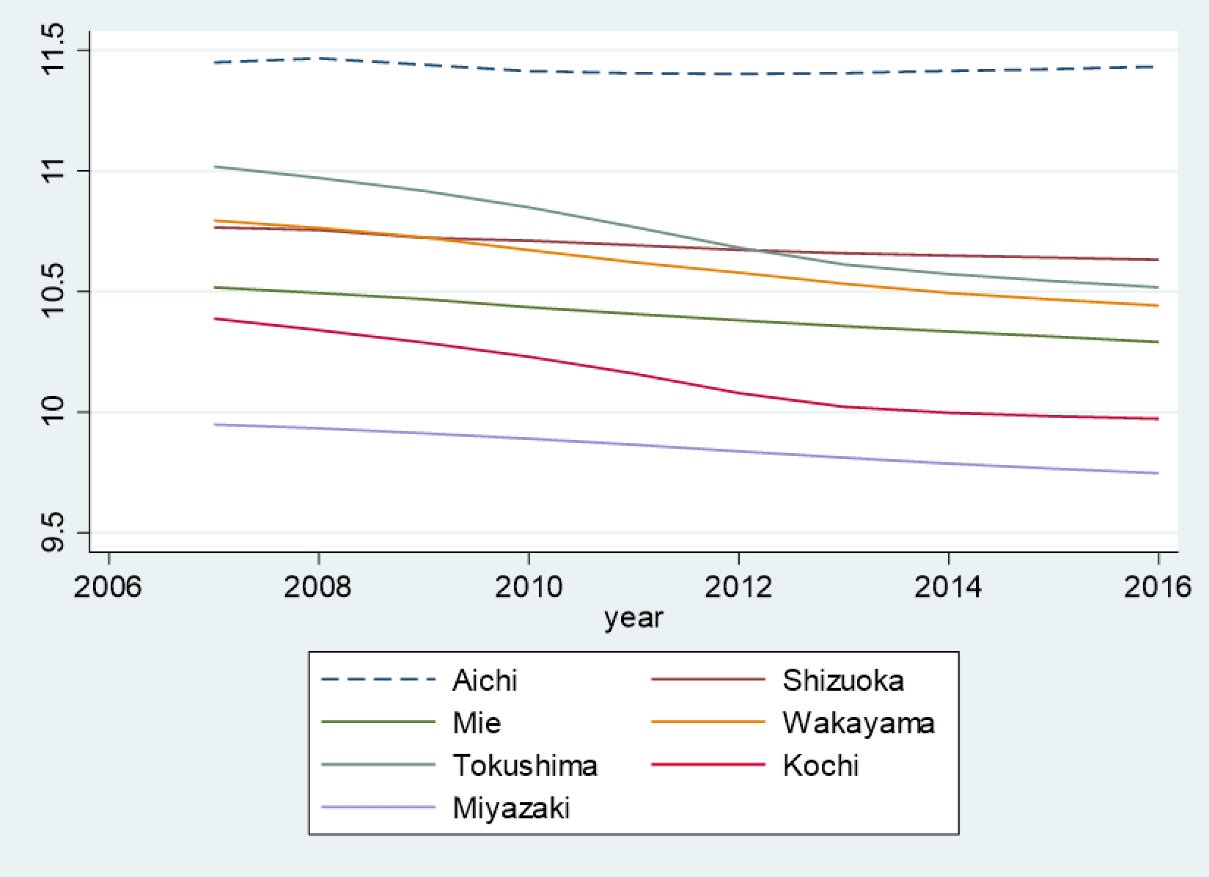


**Table A3. DDD Estimation Results** **for the Residential Areas.**

| Distance from the coastline | |  |  |  |  |
| --- | --- | --- | --- | --- | --- |
|  | 3.58 km to 6.91 km | -0.103*** | -0.0685*** | 0.00933 |  |
|  |  | (0.0270) | (0.0209) | (0.00536) |  |
|  | 1.46 km to 3.58 km | -0.00884 | 0.0140 | 0.0372** |  |
|  |  | (0.0366) | (0.0155) | (0.0119) |  |
|  | within 1.46 km | -0.117*** | -0.0834** | -0.0570* |  |
|  |  | (0.0303) | (0.0357) | (0.0276) |  |
|  |  | less than 3.6m | 3.6 to 8.8 m | 8.8 to 26.3 m |  |
|  |  |  |  |  | Elevation |

Note: The table shows the estimated $\beta_{1jk}$ in equation (2). ***, **, and * indicate that the estimated coefficient is significant at the 0.01, 0.05, and 0.10 levels, respectively. [12]’s standard error are reported in parentheses. The set of explanatory variables used in the model is identical to that of Table 4 except for regulation dummies. We control after-treatment dummy, standard site’s fixed effect, and linear time trend. Moreover, we estimate the coefficients $\beta_{2j}$ and $\beta_{3j}$ in equation (2). But they are omitted from the result.

**Table A4. DDD Estimation Results by Including Aichi Prefecture.**

| Distance from the coast line | |  |  |  |  |
| --- | --- | --- | --- | --- | --- |
|  | 3.58 km to 6.91 km | 0.00641 | -0.0696*** | -0.0427*** |  |
|  |  | (0.0105) | (0.0164) | (0.0125) |  |
|  | 1.46 km to 3.58 km | -0.0709*** | -0.0620*** | -0.0351*** |  |
|  |  | (0.0143) | (0.0138) | (0.00747) |  |
|  | within 1.46 km | -0.0630*** | -0.0654*** | -0.0283** |  |
|  |  | (0.0151) | (0.0172) | (0.00922) |  |
|  |  | less than 3.6m | 3.6 to 8.8 m | 8.8 to 26.3 m |  |
|  |  |  |  |  | Elevation |

Note: The table shows the estimated $\beta_{1jk}$ in equation (2). ***, **, and * indicate that the estimated coefficient is significant at the 0.01, 0.05, and 0.10 levels, respectively. [12]’s standard error are reported in parentheses. The set of explanatory variables used in the model is identical to that of Table 4. We control after-treatment dummy, land fixed effect, and linear time trend. Moreover, we estimate the coefficients $\beta_{2j}$ and $\beta_{3j}$ in equation (2). But they are omitted from the result.
