# Supplementary material for: The impact of Tsunamis on land appraisals: Evidence from Western Japan
Source: PLoS One. 2021 Apr 6;16(4):e0248860. doi: 10.1371/journal.pone.0248860 (PMC8023538; doi:10.1371/journal.pone.0248860)
Supplement: S1 Table — DID Estimation Result: Elevation and Appraised Land Price. (DOCX) [file pone.0248860.s002.docx]

**S1 Table. Estimation Result of All Control Variables in Table 2.** DID Estimation Result: Elevation and Appraised Land Price.

|  | (1) |
| --- | --- |
| Variables | DID |
|  |  |
| After | -0.00692 |
|  | (0.00641) |
| After × elevation less than 3.6 m | -0.0645*** |
|  | (0.0108) |
| After × elevation 3.6 m to 8.8 m | -0.0477*** |
|  | (0.0106) |
| After × elevation 8.8 m to 26.3 m | 0.0223** |
|  | (0.00755) |
| Acreage of the land | -6.09e-05 |
|  | (3.63e-05) |
| Distance from the closest major traffic facilities | 1.02e-06* |
|  | (5.44e-07) |
| Number of floors above ground | -0.339 |
|  | (0.209) |
| Building coverage ratio | -0.00208 |
|  | (0.00146) |
| Floor area ratio | 0.000135 |
|  | (0.000171) |
| Residential area | -0.00141 |
|  | (0.00258) |
| Commercial area | -0.00519 |
|  | (0.00649) |
| Industrial area | -0.0237** |
|  | (0.00814) |
| Quasi-industrial area | -0.0282** |
|  | (0.00948) |
| Supply of gas | 0.0271*** |
|  | (0.00774) |
| Supply of Sewer | -0.0125*** |
|  | (0.00316) |
| Trend | -0.0269*** |
|  | (0.00349) |
| $\mathrm{Trend}^{2}$ | 0.000397 |
|  | (0.000240) |
| Constant | 12.25*** |
|  | (0.666) |
|  |  |
| Observations | 11,624 |
| Number of standard sites | 1,166 |
| R-squared | 0.234 |
| [12]’s standard errors in parentheses |  |
| *** p<0.01, ** p<0.05, * p<0.1 |  |
