# Supplementary material for: The impact of Tsunamis on land appraisals: Evidence from Western Japan
Source: PLoS One. 2021 Apr 6;16(4):e0248860. doi: 10.1371/journal.pone.0248860 (PMC8023538; doi:10.1371/journal.pone.0248860)
Supplement: S2 Table — DID Estimation Result: Distance from the Coastline and Appraised Land Price. (DOCX) [file pone.0248860.s003.docx]

**S2 Table. Estimation Result of All Control Variables in Table 3.** DID Estimation Result: Distance from the Coastline and Appraised Land Price.

|  | (1) |
| --- | --- |
| Variables | DID |
|  |  |
| After | -0.0185* |
|  | (0.00894) |
| After × distance less than 1.46 km | -0.0710*** |
|  | (0.0156) |
| After × distance 1.46 km to 3.58 km | 0.0117 |
|  | (0.0118) |
| After × distance 3.58 km to 6.91 km | 0.0166** |
|  | (0.00667) |
| Acreage of the land | -2.67e-05 |
|  | (3.73e-05) |
| Distance from the closest major traffic facilities | -5.11e-07* |
|  | (2.47e-07) |
| Number of floors above ground | -0.335 |
|  | (0.207) |
| Building coverage ratio | -0.00328 |
|  | (0.00235) |
| Floor area ratio | 0.000105 |
|  | (0.000153) |
| Residential area | -0.000524 |
|  | (0.00260) |
| Commercial area | -0.0120* |
|  | (0.00549) |
| Industrial area | -0.00990* |
|  | (0.00497) |
| Quasi-industrial area | -0.0212** |
|  | (0.00753) |
| Supply of gas | 0.0292** |
|  | (0.00952) |
| Supply of Sewer | -0.0108*** |
|  | (0.00236) |
| Trend | -0.0270*** |
|  | (0.00349) |
| $\mathrm{Trend}^{2}$ | 0.000401 |
|  | (0.000241) |
| Constant | 12.31*** |
|  | (0.638) |
|  |  |
| Observations | 11,624 |
| Number of standard sites | 1,166 |
| R-squared | 0.234 |
| [12]’s standard errors in parentheses parentheses |  |
| *** p<0.01, ** p<0.05, * p<0.1 |  |
