# Supplementary material for: The impact of Tsunamis on land appraisals: Evidence from Western Japan
Source: PLoS One. 2021 Apr 6;16(4):e0248860. doi: 10.1371/journal.pone.0248860 (PMC8023538; doi:10.1371/journal.pone.0248860)
Supplement: S3 Table — DDD Estimation Result. (DOCX) [file pone.0248860.s004.docx]

**S3 Table. Estimation Result of All Control Variables in Table 4.** DDD Estimation Result.

|  | (1) |
| --- | --- |
| Variables | DDD |
|  |  |
| After | -0.0183* |
|  | (0.00896) |
| After × distance less than 1.46 km × elevation less than 3.6 m | -0.109*** |
|  | (0.0320) |
| After × distance 1.46 km to 3.58 km × elevation less than 3.6 m | -0.0565*** |
|  | (0.0171) |
| After × distance 3.58 km to 6.91 km × elevation less than 3.6 m | -0.0901*** |
|  | (0.0233) |
| After × distance less than 1.46 km × elevation 3.6 m to 8.8 m | -0.0516 |
|  | (0.0303) |
| After × distance 1.46 km to 3.58 km × elevation 3.6 m to 8.8 m | 0.0297* |
|  | (0.0155) |
| After × distance 3.58 km to 6.91 km × elevation 3.6 m to 8.8 m | -0.0426** |
|  | (0.0169) |
| After × distance less than 1.46 km × elevation 8.8 m to 26.3 m | -0.0941** |
|  | (0.0304) |
| After × distance 1.46 km to 3.58 km × elevation 8.8 m to 26.3 m | 0.00617 |
|  | (0.00421) |
| After × distance 3.58 km to 6.91 km × elevation 8.8 m to 26.3 m | -0.0103** |
|  | (0.00405) |
| After × distance less than 1.46 km | 0.0312* |
|  | (0.0138) |
| After × distance 1.46 km to 3.58 km | 0.0349*** |
|  | (0.0102) |
| After × distance 3.58 km to 6.91 km | 0.0552*** |
|  | (0.0151) |
| After × elevation less than 3.6 m | -0.00376 |
|  | (0.00331) |
| After × elevation 3.6 m to 8.8 m | -0.0535*** |
|  | (0.0144) |
| After × elevation 8.8 m to 26.3 m | 0.0101* |
|  | (0.00465) |
| Acreage of the land | -5.18e-05 |
|  | (3.75e-05) |
| Distance from the closest major traffic facilities | -3.98e-07* |
|  | (1.93e-07) |
| Number of floors above ground | -0.345 |
|  | (0.208) |
| Building coverage ratio | -0.00180 |
|  | (0.00143) |
| Floor area ratio | 0.000131 |
|  | (0.000161) |
| Residential area | -0.00455 |
|  | (0.00338) |
| Commercial area | -0.00755 |
|  | (0.00588) |
| Industrial area | -0.0297*** |
|  | (0.00886) |
| Quasi-industrial area | -0.0294** |
|  | (0.00924) |
| Supply of gas | 0.0253** |
|  | (0.00849) |
| Supply of Sewer | -0.0106*** |
|  | (0.00260) |
| Trend | -0.0269*** |
|  | (0.00349) |
| $\mathrm{Trend}^{2}$ | 0.000400 |
|  | (0.000240) |
| Constant | 12.25*** |
|  | (0.659) |
|  |  |
| Observations | 11,624 |
| Number of standard sites | 1,166 |
| R-squared | 0.242 |
| [12]’s standard errors in parentheses |  |
| *** p<0.01, ** p<0.05, * p<0.1 |  |
