# Supplementary material for: The impact of Tsunamis on land appraisals: Evidence from Western Japan
Source: PLoS One. 2021 Apr 6;16(4):e0248860. doi: 10.1371/journal.pone.0248860 (PMC8023538; doi:10.1371/journal.pone.0248860)
Supplement: S4 Table — DDD Estimation Results Western Japan Sample without Tsunami Risk. (DOCX) [file pone.0248860.s005.docx]

**S4 Table. Estimation Result of All Control Variables in Table A1.** DDD Estimation Results Western Japan Sample without Tsunami Risk.

|  | (1) |
| --- | --- |
| Variables | DDD |
|  |  |
| After | -0.0254** |
|  | (0.0108) |
| After × distance less than 1.46 km × elevation less than 3.6 m | 0.0119 |
|  | (0.00822) |
| After × distance 1.46 km to 3.58 km × elevation less than 3.6 m | -0.0274* |
|  | (0.0122) |
| After × distance 3.58 km to 6.91 km × elevation less than 3.6 m | -0.00658 |
|  | (0.0129) |
| After × distance less than 1.46 km × elevation 3.6 m to 8.8 m | -0.00495 |
|  | (0.00440) |
| After × distance 1.46 km to 3.58 km × elevation 3.6 m to 8.8 m | -0.0360*** |
|  | (0.00760) |
| After × distance 3.58 km to 6.91 km × elevation 3.6 m to 8.8 m | 0.0167*** |
|  | (0.00465) |
| After × distance less than 1.46 km × elevation 8.8 m to 26.3 m | -0.0137*** |
|  | (0.00411) |
| After × distance 1.46 km to 3.58 km × elevation 8.8 m to 26.3 m | -0.0304*** |
|  | (0.00643) |
| After × distance 3.58 km to 6.91 km × elevation 8.8 m to 26.3 m | -0.0272*** |
|  | (0.00794) |
| After × distance less than 1.46 km | -0.0140** |
|  | (0.00493) |
| After × distance 1.46 km to 3.58 km | 0.0520*** |
|  | (0.0114) |
| After × distance 3.58 km to 6.91 km | 0.0193** |
|  | (0.00694) |
| After × elevation less than 3.6 m | -0.0249** |
|  | (0.00849) |
| After × elevation 3.6 m to 8.8 m | -0.00866** |
|  | (0.00330) |
| After × elevation 8.8 m to 26.3 m | 0.0234*** |
|  | (0.00631) |
| Acreage of the land | -6.05e-06 |
|  | (3.42e-06) |
| Distance from the closest major traffic facilities | -4.56e-07 |
|  | (8.83e-07) |
| Number of floors above ground | -0.0427 |
|  | (0.0387) |
| Building coverage ratio | -0.00646** |
|  | (0.00239) |
| Floor area ratio | 0.00225*** |
|  | (0.000693) |
| Residential area | 0.0700 |
|  | (0.0455) |
| Commercial area | -0.000620 |
|  | (0.0269) |
| Industrial area | 0.0498 |
|  | (0.0433) |
| Quasi-industrial area | 0.0581 |
|  | (0.0435) |
| Supply of gas | 0.000254 |
|  | (0.00181) |
| Supply of Sewer | -0.0384*** |
|  | (0.00900) |
| Trend | -0.0377*** |
|  | (0.00482) |
| $\mathrm{Trend}^{2}$ | 0.00140*** |
|  | (0.000333) |
| Constant | 11.25*** |
|  | (0.106) |
|  |  |
| Observations | 51,767 |
| Number of standard sites | 5,189 |
| R-squared | 0.213 |
| [12]’s standard errors in parentheses parentheses |  |
| *** p<0.01, ** p<0.05, * p<0.1 |  |
