# Supplementary material for: The impact of Tsunamis on land appraisals: Evidence from Western Japan
Source: PLoS One. 2021 Apr 6;16(4):e0248860. doi: 10.1371/journal.pone.0248860 (PMC8023538; doi:10.1371/journal.pone.0248860)
Supplement: S5 Table — DDD Estimation Results with a Different Sample Period. (DOCX) [file pone.0248860.s006.docx]

**S5 Table. Estimation Result of All Control Variables in Table A2.** DDD Estimation Results with a Different Sample Period.

|  | (1) |
| --- | --- |
| Variables | DDD |
|  |  |
| After | -0.0101** |
|  | (0.00242) |
| After × distance less than 1.46 km × elevation less than 3.6 m | -0.0347** |
|  | (0.00771) |
| After × distance 1.46 km to 3.58 km × elevation less than 3.6 m | 0.0103 |
|  | (0.0134) |
| After × distance 3.58 km to 6.91 km × elevation less than 3.6 m | -0.0434** |
|  | (0.0105) |
| After × distance less than 1.46 km × elevation 3.6 m to 8.8 m | 0.0214*** |
|  | (0.00449) |
| After × distance 1.46 km to 3.58 km × elevation 3.6 m to 8.8 m | 0.0388** |
|  | (0.0107) |
| After × distance 3.58 km to 6.91 km × elevation 3.6 m to 8.8 m | 0.00295* |
|  | (0.00116) |
| After × distance less than 1.46 km × elevation 8.8 m to 26.3 m | -0.0311** |
|  | (0.00909) |
| After × distance 1.46 km to 3.58 km × elevation 8.8 m to 26.3 m | 0.0155** |
|  | (0.00392) |
| After × distance 3.58 km to 6.91 km × elevation 8.8 m to 26.3 m | 0.00550* |
|  | (0.00249) |
| After × distance less than 1.46 km | 0.00494 |
|  | (0.00266) |
| After × distance 1.46 km to 3.58 km | -0.0442** |
|  | (0.0101) |
| After × distance 3.58 km to 6.91 km | 0.00350 |
|  | (0.00192) |
| After × elevation less than 3.6 m | 0.00833* |
|  | (0.00311) |
| After × elevation 3.6 m to 8.8 m | 0.0181*** |
|  | (0.00358) |
| After × elevation 8.8 m to 26.3 m | 0.0228** |
|  | (0.00711) |
| Acreage of the land | -7.49e-05 |
|  | (9.21e-05) |
| Distance from the closest major traffic facilities | 1.67e-05** |
|  | (5.14e-06) |
| Number of floors above ground | -1.096** |
|  | (0.254) |
| Building coverage ratio | 0.000204 |
|  | (0.000562) |
| Floor area ratio | -0.000127** |
|  | (3.46e-05) |
| Supply of gas | 0.000100 |
|  | (0.00113) |
| Supply of Sewer | -0.0104*** |
|  | (0.00152) |
| Trend | -0.00358 |
|  | (0.00181) |
| $\mathrm{Trend}^{2}$ | -0.00330*** |
|  | (0.000307) |
| Constant | 14.48*** |
|  | (0.784) |
|  |  |
| Observations | 5,813 |
| Number of standard sites | 1,166 |
| R-squared | 0.131 |
| [12]’s standard errors in parentheses |  |
| *** p<0.01, ** p<0.05, * p<0.1 |  |
