# Supplementary material for: The impact of Tsunamis on land appraisals: Evidence from Western Japan
Source: PLoS One. 2021 Apr 6;16(4):e0248860. doi: 10.1371/journal.pone.0248860 (PMC8023538; doi:10.1371/journal.pone.0248860)
Supplement: S6 Table — DDD Estimation results for the Residential Areas. (DOCX) [file pone.0248860.s007.docx]

**S6 Table. Estimation Result of All Control Variables in Table A3.** DDD Estimation results for the Residential Areas.

|  | (1) |
| --- | --- |
| Variables | DDD |
|  |  |
| After | -0.0191 |
|  | (0.0123) |
| After × distance less than 1.46 km × elevation less than 3.6 m | -0.117*** |
|  | (0.0303) |
| After × distance 1.46 km to 3.58 km × elevation less than 3.6 m | -0.00884 |
|  | (0.0366) |
| After × distance 3.58 km to 6.91 km × elevation less than 3.6 m | -0.103*** |
|  | (0.0270) |
| After × distance less than 1.46 km × elevation 3.6 m to 8.8 m | -0.0834** |
|  | (0.0357) |
| After × distance 1.46 km to 3.58 km × elevation 3.6 m to 8.8 m | 0.0140 |
|  | (0.0155) |
| After × distance 3.58 km to 6.91 km × elevation 3.6 m to 8.8 m | -0.0685*** |
|  | (0.0209) |
| After × distance less than 1.46 km × elevation 8.8 m to 26.3 m | -0.0570* |
|  | (0.0276) |
| After × distance 1.46 km to 3.58 km × elevation 8.8 m to 26.3 m | 0.0372** |
|  | (0.0119) |
| After × distance 3.58 km to 6.91 km × elevation 8.8 m to 26.3 m | 0.00933 |
|  | (0.00536) |
| After × distance less than 1.46 km | 0.0176 |
|  | (0.0117) |
| After × distance 1.46 km to 3.58 km | 0.0296** |
|  | (0.00995) |
| After × distance 3.58 km to 6.91 km | 0.0555** |
|  | (0.0184) |
| After × elevation less than 3.6 m | -0.00230 |
|  | (0.00456) |
| After × elevation 3.6 m to 8.8 m | -0.0283** |
|  | (0.0108) |
| After × elevation 8.8 m to 26.3 m | -0.0123*** |
|  | (0.00283) |
| Acreage of the land | -0.00261* |
|  | (0.00117) |
| Distance from the closest major traffic facilities | -2.89e-06*** |
|  | (7.38e-07) |
| Number of floors above ground | -0.766 |
|  | (0.421) |
| Building coverage ratio | -0.0135*** |
|  | (0.00217) |
| Floor area ratio | 0.00291*** |
|  | (0.000672) |
| Supply of gas | 0.0135 |
|  | (0.0115) |
| Supply of Sewer | -0.0156*** |
|  | (0.00350) |
| Trend | -0.0216*** |
|  | (0.00360) |
| $\mathrm{Trend}^{2}$ | 0.000181 |
|  | (0.000218) |
| Constant | 14.04*** |
|  | (1.625) |
|  |  |
| Observations | 7,781 |
| Number of standard sites | 848 |
| R-squared | 0.192 |
| [12]’s standard errors in parentheses |  |
| *** p<0.01, ** p<0.05, * p<0.1 |  |
