# Supplementary material for: The impact of Tsunamis on land appraisals: Evidence from Western Japan
Source: PLoS One. 2021 Apr 6;16(4):e0248860. doi: 10.1371/journal.pone.0248860 (PMC8023538; doi:10.1371/journal.pone.0248860)
Supplement: S7 Table — DDD Estimation Results by Including Aichi Prefecture. (DOCX) [file pone.0248860.s008.docx]

**S7 Table. Estimation Result of All Control Variables in Table A4.** DDD Estimation Results by Including Aichi Prefecture.

|  | (1) |
| --- | --- |
| Variables | DDD |
|  |  |
| After | -0.00246 |
|  | (0.00813) |
| After × distance less than 1.46 km × elevation less than 3.6 m | -0.0630*** |
|  | (0.0151) |
| After × distance 1.46 km to 3.58 km × elevation less than 3.6 m | -0.0709*** |
|  | (0.0143) |
| After × distance 3.58 km to 6.91 km × elevation less than 3.6 m | 0.00641 |
|  | (0.0105) |
| After × distance less than 1.46 km × elevation 3.6 m to 8.8 m | -0.0654*** |
|  | (0.0172) |
| After × distance 1.46 km to 3.58 km × elevation 3.6 m to 8.8 m | -0.0620*** |
|  | (0.0138) |
| After × distance 3.58 km to 6.91 km × elevation 3.6 m to 8.8 m | -0.0696*** |
|  | (0.0164) |
| After × distance less than 1.46 km × elevation 8.8 m to 26.3 m | -0.0283** |
|  | (0.00922) |
| After × distance 1.46 km to 3.58 km × elevation 8.8 m to 26.3 m | -0.0351*** |
|  | (0.00747) |
| After × distance 3.58 km to 6.91 km × elevation 8.8 m to 26.3 m | -0.0427*** |
|  | (0.0125) |
| After × distance less than 1.46 km | -0.0419*** |
|  | (0.00891) |
| After × distance 1.46 km to 3.58 km | 0.000313 |
|  | (0.00399) |
| After × distance 3.58 km to 6.91 km | 0.0224** |
|  | (0.00749) |
| After × elevation less than 3.6 m | 0.0120** |
|  | (0.00378) |
| After × elevation 3.6 m to 8.8 m | 0.0302*** |
|  | (0.00738) |
| After × elevation 8.8 m to 26.3 m | 0.0438*** |
|  | (0.0105) |
| Acreage of the land | 1.15e-05 |
|  | (1.57e-05) |
| Distance from the closest major traffic facilities | 1.99e-07 |
|  | (7.18e-07) |
| Number of floors above ground | -0.129 |
|  | (0.0822) |
| Building coverage ratio | -0.00610* |
|  | (0.00289) |
| Floor area ratio | 0.000168* |
|  | (8.93e-05) |
| Residential area | -0.0284*** |
|  | (0.00868) |
| Commercial area | -0.0725*** |
|  | (0.0141) |
| Industrial area | -0.0718*** |
|  | (0.0189) |
| Quasi-industrial area | -0.0677*** |
|  | (0.0158) |
| Supply of gas | 0.00769** |
|  | (0.00318) |
| Supply of Sewer | -0.0125** |
|  | (0.00402) |
| Trend | -0.0274*** |
|  | (0.00596) |
| ${\sum{Trend}^{2}}$ | 0.00134*** |
|  | (0.000388) |
| Constant | 12.19*** |
|  | (0.255) |
|  |  |
| Observations | 23,221 |
| Number of standard sites | 2,330 |
| R-squared | 0.1771 |
| [12]’s standard errors in parentheses |  |
| *** p<0.01, ** p<0.05, * p<0.1 |  |
|  |  |
